# Impact of Advanced Footwear Technology on Running Economy at Slower Running Speeds: A Randomised, Cross-Over Investigation

**DOI:** 10.1186/s40798-026-00977-3

**Published:** 2026-02-17

**Authors:** Aline Bolliger, Christina M. Spengler, Fernando G. Beltrami

**Affiliations:** Exercise Physiology Lab, Institute of Human Movement Sciences and Sport, ETH Zurich, Gloriastrasse 37/39, 8092 Zurich, Switzerland

**Keywords:** Footwear, Super shoes, Marathon, Recreational runners

## Abstract

**Background:**

Compared with traditional running shoes, Advanced Footwear Technology (AFT) improves the oxygen cost of running (O_2_ cost) for athletes competing at fast speeds. Less clear are the effects of these modern shoes at the slower speeds commonly adopted by recreational runners. Therefore, this study’s primary aim was to assess the effects of AFT shoes on O_2_ cost at slow running speeds.

**Methods:**

Fourteen moderately-trained runners (6 men, 8 women, age 25.5 ± 2.8 years, body mass index 21.7 ± 2.3 kg m^−2^, V̇O_2peak_ 49.8 ± 5.1 ml O_2_ kg^−1^ min^−1^) ran at four speeds (7.5, 9.0, 10.5 and 12.0 km h^−1^) with three different footwear conditions: traditional running shoes (On Cloudrunner 2, TRA); AFT shoes (On Cloudboom Echo 3, CBE); and prototype shoes combining AFT and standard features (high compliance, energy return, and mass; On Prototype, MIX). The full protocol was repeated on three different days by each participant.

**Results:**

Combining all tested speeds, O_2_ cost was lower for CBE compared with both TRA (−5.4 ml O_2_ kg^−1^ km^−1^, 95% CI: −6.9 to −3.9 ml O_2_ kg^−1^ km ^−1^, *p* < 0.001) and MIX (−4.1 ml O_2_ kg^−1^ km^−1^, 95% CI: –−5.6 to −2.6 ml O_2_ kg^−1^ km ^−1^, *p* < 0.001), whereas no difference between MIX and TRA could be detected (95% CI: −3.4 to 0.8 ml O_2_ kg^−1^ km^−1^, *p* = 0.269). Differences in O_2_ cost between shoes were independent of speed. Perceived effort was lower for CBE compared with TRA only (−0.2 points, 95% CI −0.4 to −0.1 points), whereas no differences were detected for perceived comfort between any of the shoes (*p* = 0.377). No clear effect of footwear was detected for cadence, ground contact time, or leg stiffness. Comparing the most and least liked shoe models revealed preference for both lower O_2_ cost and higher comfort, with larger effect sizes for comfort.

**Conclusion:**

AFT shoes can provide meaningful metabolic savings even at very low running speeds, with no distinguishable speed-dependence, which cannot be explained by changes in spatiotemporal variables. AFT shoes for recreational runners should be further improved to maintain metabolic efficiency while not sacrificing perceived comfort, which is a stronger determinant of shoe preference.

**Supplementary Information:**

The online version contains supplementary material available at 10.1186/s40798-026-00977-3.

## Keypoints

- Early research suggests that improvements in running economy from advanced footwear technology shoes are limited to high running speeds.
- We demonstrate that advanced footwear technology shoes improve running economy in speeds ranging from 7.5 to 12.0 km h^−1^ in moderately trained runners, with no discernible speed-dependent effect.
- We further show that shoes prioritizing energy return and softness over shoe mass led to a decrement in running economy in this population compared with advanced footwear technology shoes, with no noticeable gains in perceived comfort.

**Supplementary Information:**

The online version contains supplementary material available at 10.1186/s40798-026-00977-3.

## Introduction

Modern running shoes, termed Advanced Footwear Technology (AFT), have revolutionized the running footwear industry by reducing the metabolic cost of running at a given speed compared with traditional running shoes [1]. AFT shoes typically combine minimal mass, highly compliant and resilient foams and increased longitudinal bending stiffness—the latter often provided by a stiff carbon fibre plate. Even though the precise mechanisms underlying the benefits of AFT remain elusive, it is believed that improvements in running economy stem from a combination of these factors [2, 3].

Several studies have investigated the effects of AFT shoes on running economy, reporting metabolic savings of ~2.7–4.2% at 13.0–18.0 km h^−1^ [1, 4–6], with no distinct speed-dependent effects found within this range. However, much of the running community consists of recreational runners [7] whose average marathon speed ranges around 8–10 km h^−1^ [8]. The effects of AFT shoes in this specific population remain relatively unexplored, with the only two studies performed to date suggesting a loss of energetic savings at lower speeds [9, 10]. In one study, metabolic savings decreased from 1.6% at 12 km h^−1^ to 0.9% at 10 km h^−1^ [9], while the second investigation reported a decreased in metabolic savings from 5.0% at an average speed of 11.5 km h^−1^ to 3.8% at an average speed of 9.4 km h^−1^ [10]. In both the latter studies, differences in running economy either failed to reach statistical significances at lower speeds [9] or were significantly lower than those at higher speeds [10]. In another investigation, AFT shoes reduced the metabolic cost compared with traditional running shoes in recreational runners at speeds ranging between 11.0 and 14.7 km h^−1^, but not when AFT shoes were compared to lightweight running flats [11]. The latter findings bring attention to a common problem in footwear research, namely that comparisons between two specific models cannot be generalized to all AFT and traditional footwear models, not least because AFT models also show variability in their effect on running economy [12]. In this context, we have previously shown that the On Cloudboom Echo 3 shoes, bearing all features of an AFT shoe, improves running economy by 2.4 ± 1.1% compared with a traditional running shoe of the same brand (On Cloudrunner) when tested at 16 km h^−1^with well-trained runners [13]. Expanding these findings to a less trained cohort at lower speeds provides an interesting possibility to directly address the speed-dependency of AFT benefits. In addition, comparing against shoes combine features of both traditional running shoes and AFT can expand the current understanding of which features contribute the most to the improved running economy of AFT shoes.

Prior research proposed two hypotheses for the loss of effect of AFT shoes on running economy at slower speeds [9]. One possibility, called the force threshold hypothesis, proposes that below a certain speed threshold the reduced vertical ground reaction forces might not completely compress the midsole foam, underutilizing the full elastic potential of the shoe. A second, non-exclusive possibility is that the increased longitudinal bending stiffness of the embedded rigid plate favours faster speeds, possibly even having deleterious effects at lower speeds. Previous studies have also reported changes in spatiotemporal parameters during running whilst wearing AFT shoes, including an increased stride length and leg stiffness coupled with a decreased in ground contact time [1, 4]. As slower running speeds can have distinct effects on spatiotemporal and kinetic characteristics during running, it is important to evaluate the impact of the altered longitudinal bending stiffness and the compliant, resilient foam of the AFT on these metrics [9]. Finally, since comfort is one the most important components of what leads to a certain shoe choice by recreational runners [14], investigation of the relationship between perceived comfort and running economy in AFT shoes is essential. Indeed, while some studies report a positive impact of perceived footwear comfort on running performance [15–17], including AFT shoes [18], others point instead towards a lack of effect of comfort on running economy [14, 19].

Increasing the current knowledge around the effects of AFT shoes at slower speeds and its interaction with other aspects of running is of great benefit to a large part of the running community. Therefore, the goal of this study was to assess the effects of AFT shoes at slower speeds in three different footwear conditions, including a traditional running shoe (On Cloudrunner 2, TRA), an AFT shoe optimized for performance at higher speeds (On Cloudboom Echo 3, CBE) and a shoe combining features of both AFT and traditional shoes (On prototype, MIX). Based on the results of previous research, we hypothesized that (1) there would still be benefits in O_2_ cost at lower speeds, but they would diminish with decreasing velocities. Furthermore, we expected differences in spatiotemporal parameters between footwear conditions at slower running speeds and hypothesized that (2) a lower cadence and ground contact time coupled with an increase in leg stiffness would be observed in AFT shoes. Besides that, as we assumed that perceived comfort could indeed be associated with improved mechanics and running economy, we expected that (3) increased comfort ratings would be associated with improved running economy.

## Methods

### General Design

This study was a randomised, cross-over trial. The participants visited the laboratory on three different occasions, each separated by 3–7 days, and the same running protocol (see below) was performed on each occasion, as a triplicate. To maintain standard physiological conditions, participants were instructed to refrain from intensive training during the 48 h preceding each test session and to avoid caffeine on the test day. A logbook was used to verify that they replicated their nutrition, sleep, and training patterns prior to each session. All three shoes were tested in a randomised and balanced order between and within participants on each of the test days, so that each shoe was tested once as the first, second, and the third condition over the three experimental visits.

### Participants

Sample size calculations (G-power 3.1 [20]), were performed assuming similar effects sizes as we recently found at higher speeds between the tested AFT and traditional shoes [13]. Calculations were performed considering a within design with 3 different shoes and four different speeds. For an alpha level of 0.05, power at 0.80, 13 participants would be required for an effect size η_p_^2^ of 0.58. To ensure robustness of the data, sixteen recreational runners were initially recruited for this study via personal contact and advertisements in running groups. Two male participants did not complete the study trials due to scheduling issues. We present the data for 6 male participants (age 24.6 ± 1.4 years; height 181.0 ± 6.2 cm; body mass 74.2 ± 5.9 kg; body fat 14.8 ± 1.6%; V̇O_2peak_ 53.5 ± 3.8 ml O_2_ kg^−1^ min^−1^) who could comfortably run in shoes EU size 44 and 8 female participants (aged 26.1 ± 3.5 years; height 162.3 ± 5.6 cm; body mass 55.9 ± 5.1 kg; body fat 25.6 ± 5.2%; V̇O_2peak_ 47.0 ± 4.2 ml O_2_ kg^−1^ min^−1^) with shoe size EU 38.5. Participants were included in the study if they reported the ability to complete a half marathon in less than 2 h 30 min but more than 1 h 30 min or a 10-km race within the range of 40 min to 1 h 10 min, and run at least twice per week in the previous 3 months. In addition, participants had to be unfamiliar with the two commercially available shoes tested. Runners with lower-limb injuries in the past 3 months or a history of cardiovascular or pulmonary diseases were excluded. This study was approved by the Ethics Committee of ETH Zurich (2023-N-116) and abided by the ethical standards of the Declaration of Helsinki, except for publication in a public database. All participants provided written informed consent before participation.

### Experimental Protocol

During visit 1, the participants were required to fill out the consent form, a questionnaire on their health status and their training situation. Additionally, their height, weight and leg length were measured using a scale (Seca 635, Seca, Hamburg, Germany, to the nearest 0.1 kg) and stadiometer (to the nearest 0.5 cm). After the completion of the running protocol (see below) and a 30-min rest period, the participants performed an incremental running test on a motorized treadmill (H/P Cosmos, Cosmos Pulsar 3p, Germany) to assess their peak aerobic capacity (V̇O_2peak_). The test started with 4 min at 8.0 km h^−1^ and thereafter the speed was increased by 1 km h^−1^ every minute until volitional exhaustion, with the gradient kept constant at 1% throughout the test. During the tests, participants breathed through a facemask connected to an ergospirometer (Metamax 3B, Cortex Biophysik, Germany) for assessment of gas exchange variables. The device was calibrated for flow (using a 3-L syringe) and gas concentration (2-point calibration using room air plus a mixture of 15% O_2_ and 5% CO_2_ in N_2_), strictly according to the manufacturer’s instructions, prior to each test. Participants also wore a heart rate monitor attached to a chest strap (Polar H10, Polar, Finland). On visit 2 the participants underwent a dual X-ray absorptiometry scan prior to the running protocol (Lunar iDXA, GE Healthcare, Madison, USA), to assess body composition and body fat percentage. On visit 3, the participants completed the running protocol a final time.

The running protocol used the same equipment as the incremental exercise test. The protocol began with a standardized incremental warm-up (2 min at 7.5 km h^−1^, 1 min at 9.0, 1 min at 10.5 km h^−1^ and 1 min at 12 km h^−1^). After a 5-min break, the test protocol started, consisting of three 14-min runs interspersed by 20 min of rest, where each of the 14-min runs was performed with one of the tested shoes. Each run started with a 5-min stage at 7.5 km h^−1^, followed by three 3-min stages at 9.0, 10.5 and 12 km h^−1^ respectively. Thirty seconds prior to the end of each stage, the participants were asked to rate their perceived effort on a modified 1–10 Borg scale. Participants were also fitted with an elastic belt worn around the waist, where an accelerometer pod was attached (±16 g range, sampling at 1024 Hz, Runeasi, Tienen, Belgium). This was used to assess spatiotemporal variables throughout the protocol (see data analysis). After completing each run, the participants were requested to fill out a shoe questionnaire to assess their experience with the tested footwear, including perceptions related to the general comfort, energy return and springiness, and the overall satisfaction. These were gathered using a standard 100-mm VAS scale and are reported in the supplementary information. At the end of each test day the participants were asked to rank the shoes according to their preference.

### Footwear Conditions

In this study, three shoe models were tested, all provided by On Running AG (Zurich, Switzerland): On Cloudrunner 2 (TRA), On prototype (MIX) and the On Cloudboom Echo 3 (CBE). The TRA is a traditional entry-level running shoe that served as the control condition for comparison. The MIX is a prototype shoe combining features of both AFT and traditional shoes; it features a rigid nylon plate along with a specialized foam double layer of polyether block amide (PEBA) on top and a blend of ethylene and vinyl acetate—thermoplastic polyester elastomer (EVA-TPEE) underneath. Serving as a high-end AFT shoe, the CBE features a carbon fibre plate coupled with a compliant and resilient foam (PEBA), along with minimal weight. A description of the mechanical properties of the different shoes is given in Table 1. The total mileage of any pair of shoes used did not exceed 80 km, to avoid possible deterioration of mechanical properties.

**Table 1:** Footwear characteristics

Shoe	Mass (g)	Bending Stiffness (N mm^−1^)	Heel				Forefoot			
			Deformation (mm)	Resilience (%)	ER (J)	Stiffness (N mm^−1^)	Deformation (mm)	Resilience (%)	ER (J)	Stiffness (N mm^−1^)
TRA	277	8.0	18.6	73.8	6.8	74.7	16.1	72.9	5.8	110.3
MIX	312	13.6	24.7	79.8	10.3	59.9	19.2	83.2	7.8	92.8
CBE	215	23.9	19.9	85.5	8.9	79.7	14.6	85.1	7.2	121.2

Data of shoe size EU 44, *ER* energy returned

### Data Analysis and Statistics

All physiological and spatiotemporal data were exported in 5-s epochs for further processing. For further statistical analysis, the initial 2 min of the running protocol was excluded. Additionally, the first 1.5 min and last 10 s of each speed stage were also excluded, to avoid non-steady state data. Data points that exceeded a difference of three times the standard deviation of each interval were considered outliers and excluded from the gas exchange data. V̇O_2peak_ was defined as the highest 30 s value measured anywhere during the incremental exercise test. As different speeds were tested in this study, the oxygen consumption (ml O_2_ kg^−1^ min^−1^) was converted to O_2_ cost, by dividing it by the running speed in km min^−1^.

Spatiotemporal data included cadence, flight time, ground contact time, flight ratio (defined as flight time divided by the sum of flight time and ground contact time), impact magnitude and impact duration (defined as the time from foot strike until peak force is reached). Based thereon, leg stiffness (in kN m^−1^) was calculated according to Morin [21]. Briefly, this method derives leg stiffness from changes in ground contact time and flight time for a given speed, mass and leg length, modelling the force–time curve by a sine function.

After an initial analysis of the differences in O_2_ cost between the different footwear conditions could not detect sex-dependent effects (performed using independent t-tests), the data from the male and female participants were pooled together for further analysis. A one-way repeated measures analysis of variance (ANOVA) was conducted to compare the gas exchange, heart rate and spatiotemporal variables between the three shoe conditions. In case of significant main effects, Tukey’s honest significant difference post hoc analysis was performed. The effect of footwear on speed was assessed by calculating the V̇O_2_ versus speed slope for each participant and footwear conditions. The individual slopes were then compared between the three footwear conditions using repeated measures ANOVA with Tukey’s post hoc. To assess the interactions between footwear and speed, two-way ANOVAs were carried out with Tukey’s post hoc where applicable. Person’s product-moment correlation as well as repeated measures correlation analysis [22] were executed to explore the relationship between comfort and O_2_ cost. Additionally, paired t-tests were performed to compare perceived comfort and O_2_ cost between the favourite and least-favourite footwear conditions on the final day of testing. The effect sizes (ES) for pairwise comparisons were calculated as the d_z_ proposed by Cohen [23], being equal to the mean difference between groups divided by the standard deviation of the change scores. The statistical significance threshold for all analyses was set at *p* ≤ 0.05. All statistical tests were performed using Prism 10.0 (Graphpad, La Jolla, US), except for the repeated measures correlation, which was performed using an online platform [24] based on the rmcorr package for R.

## Results

### Physiological Variables

**Fig. 1:**
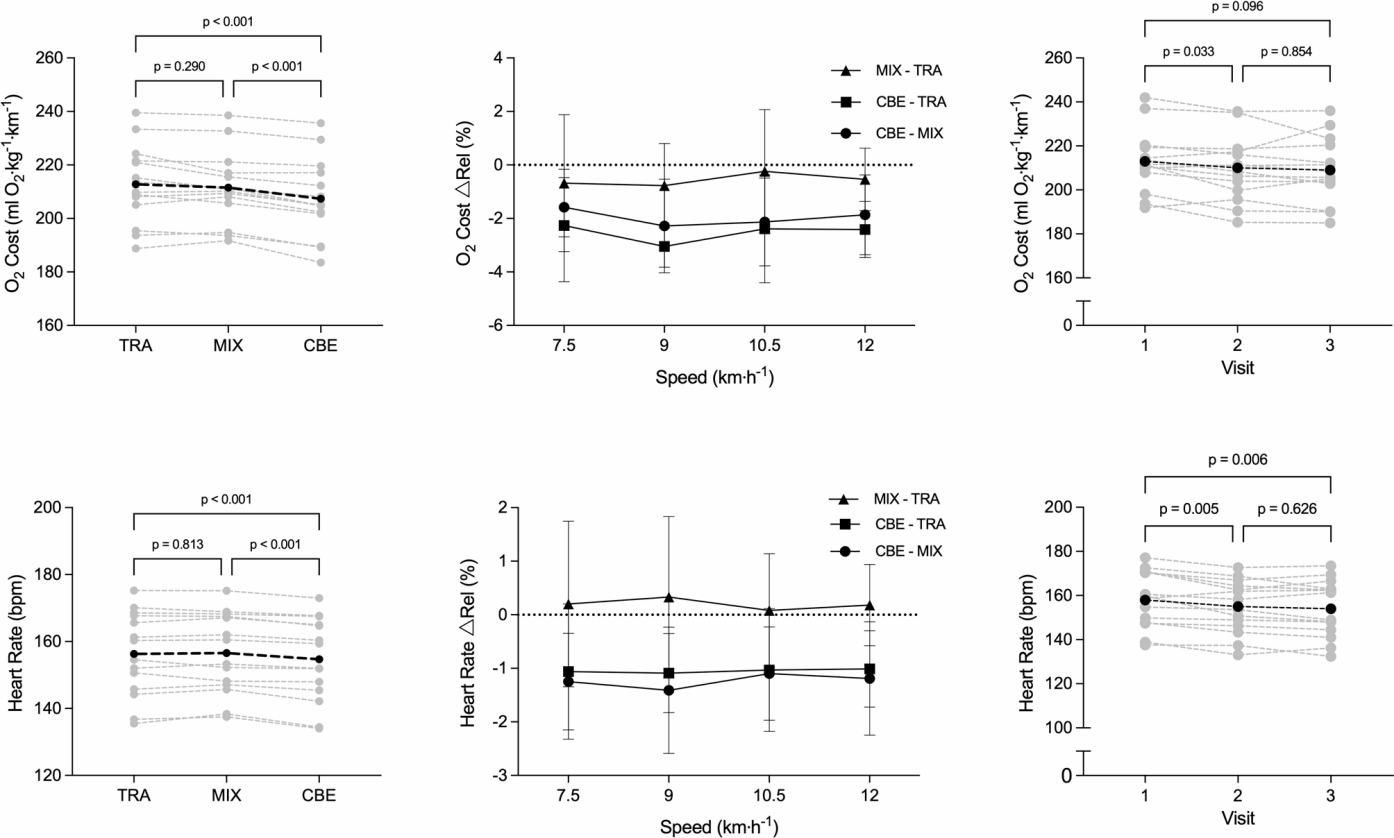
*Left* Comparison of O_2_ cost (*top*) and heart rate (*bottom*) between footwear condition as the mean of all three test days and speeds. *Centre* Average ± standard deviation of relative delta between shoes for O_2_ cost (*top*) and heart rate (*bottom*) over the speeds tested. *Right* Progression of O2 cost (*top*) and heart rate (*bottom*) over the three study visits. *Grey lines* are individual values; *black line* is average. TRA = On Cloudrunner 2; MIX = On Prototype; CBE = On Cloudboom Echo 3

The average O_2_ cost for each footwear condition with the individual responses, as well as the relative rates of change over the speed stages and their inter-trial variability are displayed in Fig. 1 and Table 2. Taking all visits and speeds into consideration, one-way ANOVA indicated a significant effect of footwear on the O_2_ cost (*p* < 0.001). Tukey’s post hoc analysis revealed a mean reduction in O_2_ cost in CBE vs. TRA of 5.4 ± 2.2 ml O_2_ kg^−1^ km^−1^ (95% CI: −6.9 to −3.9 ml O_2_ kg^−1^ km ^−1^, *p* < 0.001, ES = 2.5) and in CBE vs. MIX of 4.1 ± 2.1 ml O_2_ kg^−1^ km ^−1^ (95% CI: −5.6 to −2.6 ml O_2_ kg^−1^ km ^−1^, *p* < 0.001, ES = 2.0), whereas no differences were detected between MIX and TRA (95% CI: −3.4 to 0.8 ml O_2_ kg^−1^ km ^−1^, *p* = 0.269, ES = 0.4). In relative terms, running economy improved when wearing the CBE by 2.5 ± 1.1% compared with TRA and by 2.0 ± 1.1% compared with MIX. No difference in the slopes of O_2_ cost over speed could be detected between the different footwear (*p* = 0.730, Fig. 1, top centre panel). Likewise, a two-way ANOVA showed no speed-shoe interaction effect (*p* = 0.681).

**Table 2:** Average ± standard deviations of physiological and perceptual variables (n = 14), as well as spatiotemporal variables (n = 13) for each of the three footwear conditions over the four speeds tested

	Speed	TRA	MIX	CBE
Physiological and perceptual variables				
O_2_ cost (ml O_2_ kg^−1^ km^−1^)	7.5	219.1 ± 18.9	217.4 ± 17.5	214.0 ± 17.4*^,£^
	9.0	214.3 ± 17.0	212.5 ± 16.3	207.8 ± 17.3*^,£^
	10.5	210.1 ± 14.1	209.5 ± 13.7	205.1 ± 15.4*^,£^
	12.0	207.3 ± 11.7	206.1 ± 10.5	202.3 ± 12.2*^,£^
Heart rate (bpm)	7.5	137.7 ± 13.6	138.5 ± 13.7	136.8 ± 13.2*^,£^
	9.0	150.9 ± 14.0	151.8 ± 13.4	149.8 ± 13.1*^,£^
	10.5	163.2 ± 13.0	163.7 ± 12.1	162.0 ± 12.8*^,£^
	12.0	173.7 ± 11.4	174.3 ± 10.5	172.4 ± 11.3*^,£^
Minute ventilation (L min^−1^)	7.5	49.0 ± 7.3	49.1 ± 7.5	48.4 ± 7.2
	9.0	59.4 ± 10.5	58.9 ± 9.9	58.3 ± 9.9
	10.5	70.7 ± 12.7	70.0 ± 11.9	69.0 ± 13.1*
	12.0	82.1 ± 15.4	82.4 ± 15.4	79.8 ± 15.2*^,£^
Perceived effort (units)	7.5	1.4 ± 0.7	1.5 ± 0.8	1.4 ± 0 0.7
	9.0	2.6 ± 1.0	2.6 ± 1.1	2.5 ± 1.1
	10.5	4.2 ± 1.6	3.9 ± 1.5*	3.9 ± 1.6*
	12.0	5.6 ± 1.9	5.3 ± 1.9*	5.1 ± 1.7*
Spatiotemporal variables				
Cadence (steps min^−1^)	7.5	158.6 ± 7.4	158.5 ± 7.6	158.5 ± 7.2
	9.0	161.8 ± 8.3	161.7 ± 8.3	161.7 ± 8.1
	10.5	165.1 ± 9.3	165.1 ± 9.2	165.5 ± 8.5
	12.0	168.9 ± 9.8	169.0 ± 9.9	169.7 ± 9.3^£^
Ground contact time (ms)	7.5	269.7 ± 23.3	269.5 ± 22.5	269.1 ± 24.7
	9.0	251.2 ± 16.9	250.3 ± 16.6	249.9 ± 17.0
	10.5	238.0 ± 14.6	238.2 ± 14.6	237.4 ± 15.7
	12.0	229.2 ± 19.7	229.1 ± 16.2	228.0 ± 16.8
Leg stiffness (kN m^−1^)	7.5	4.1 ± 1.1	4.1 ± 1.1	4.2 ± 1.1
	9.0	5.0 ± 1.2	5.1 ± 1.1	5.2 ± 1.2
	10.5	5.8 ± 1.4	5.9 ± 1.2	5.9 ± 1.3
	12.0	6.6 ± 2.5	6.5 ± 1.5	6.6 ± 1.6
Flight ratio (%)	7.5	28.8 ± 5.2	28.9 ± 5.3	29.0 ± 6.2
	9.0	32.4 ± 4.0	32.7 ± 3.9	32.8 ± 4.3
	10.5	34.6 ± 3.8	34.6 ± 3.8	34.6 ± 4.2
	12.0	35.6 ± 5.0	35.6 ± 4.5	35.6 ± 4.8
Impact magnitude (g)	7.5	4.1 ± 1.6	4.0 ± 1.5	4.2 ± 1.6
	9.0	4.5 ± 1.7	4.4 ± 1.6	4.5 ± 1.7
	10.5	4.8 ± 1.7	4.6 ± 1.6	4.7 ± 1.6
	12.0	5.0 ± 1.7	4.9 ± 1.5	5.0 ± 1.5
Impact duration (ms)	7.5	62.2 ± 20.7	60.5 ± 19.1	59.8 ± 19.7*
	9.0	59.2 ± 19.2	56.8 ± 16.3	57.4 ± 17.8*
	10.5	56.7 ± 18.5	55.8 ± 16.5	56.9 ± 18.1
	12.0	55.1 ± 21.0	55.8 ± 19.1	56.8 ± 19.7

TRA = On Cloudrunner 2; MIX = On Prototype; CBE = On Cloudboom Echo 3

^*^indicates significant difference from TRA

^£^indicates significant difference from MIX.

A significant effect of footwear could also be detected for heart rate (*p* < 0.001). Tukey’s post hoc analysis revealed a mean reduction in CBE vs. TRA of 1.6 ± 1.0 bpm (95% CI: −2.3 to −0.9 bpm, *p* < 0.001) and in CBE vs. MIX of 1.9 ± 1.2 bpm (95% CI: −2.7 to −1.1 bpm, *p* = 0.001), whereas no differences were detected between MIX and TRA (95% CI: −0.8 to 1.3 bpm, *p* = 0.813). No difference in the slopes of heart rate over speed could be detected between the different footwear (*p* = 0.925, Fig. 1, bottom centre panel). Likewise, a two-way ANOVA showed no speed-shoe interaction effect (*p* = 0.734).

Minute ventilation showed a similar pattern as O_2_ cost and heart rate, with lower values for CBE compared with both TRA (95% CI −2.4 to −0.4 L min^−1^, *p* = 0.007) and MIX (−1.9 to 0.5 L min^−1^, *p* = 0.001), whereas no differences could be detected between MIX and TRA (*p* = 0.786).

### Effects of Familiarization and Trial Number on O_2_ Cost and Heart Rate

When pooling all footwear together, a difference in O_2_ cost (*p* = 0.036) between the different test days was indicated by one-way ANOVA. Subsequent Tukey’s post hoc comparisons revealed a significant reduction of O_2_ cost between the first and second test days of 3.4 ± 4.4 ml O_2_ kg^−1^ km^−1^ (95% CI: −6.5 to −0.26 ml O_2_ kg^−1^ km^−1^, *p* = 0.033, ES = 0.8).

Analysis of the O_2_ cost of the first test day alone, ignoring the repeated trials on visits 2 and 3, would show a significant effect of footwear (*p* = 0.009). Tukey’s post hoc analysis indicated a significant reduction in O_2_ cost between CBE vs. TRA (−6.4 ± 4.6 ml O_2_ kg^−1^ km^−1^; 95% CI: −9.6 to −3.2 ml O_2_ kg^−1^ km^−1^, *p* < 0.001, ES = 0.5), corresponding to a relative reduction of 2.9 ± 2.0% in O_2_ cost. For CBE vs. MIX (*p* = 0.308) or MIX vs. TRA (*p* = 0.192), no statistical differences would be detected. No differences would be detected for the slopes of O_2_ cost over speeds for the different footwear on day 1 alone (p = 0.900). With regards to heart rate, when all footwear were pooled together to investigate between-day differences, there was a reduction in heart rate of 3.4 ± 3.3 bpm from the first to the second test day (95% CI: −5.8 to −1.1 bpm, *p* = 0.005) and of 4.2 ± 4.1 bpm from the first to the third (95% CI: −7.1 to −1.3 bpm, *p* = 0.006). No differences in heart rate could be detected between the second and third day of testing (95% CI −1.4 to 3.6 bpm, *p* = 0.626).

### Perceptual Variables

**Fig. 2:**
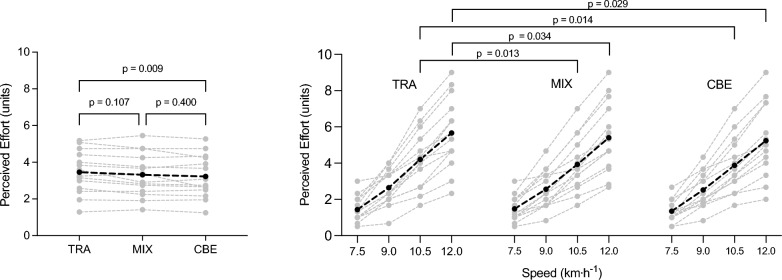
*Left* rating of perceived effort on a modified (1–10) Borg Scale averaged over the different speeds. *Right* rating of perceived effort for each footwear condition and speed. *Grey lines* are individual values; *black line* is average. TRA = On Cloudrunner 2; MIX = On Prototype; CBE = On Cloudboom Echo 3

The individual and average perceived effort ratings across footwear conditions and speeds are depicted in Fig. 2. A significant effect of footwear on perceived effort was detected (*p* = 0.006), with CBE showing 0.2 ± 0.2 units less perceived effort compared with TRA (95% CI: −0.4 to −0.1 units, *p* = 0.009). Tukey’s multiple comparisons test revealed higher perceived effort (~0.3 to 0.4 units) for TRA compared with both MIX and CBE at 10.5 and 12.0 km h^−1^ (all *p* ≤ 0.029). At the lower two speeds, no differences were detected between the different footwear conditions (all *p* ≥ 0.260).

**Fig. 3:**
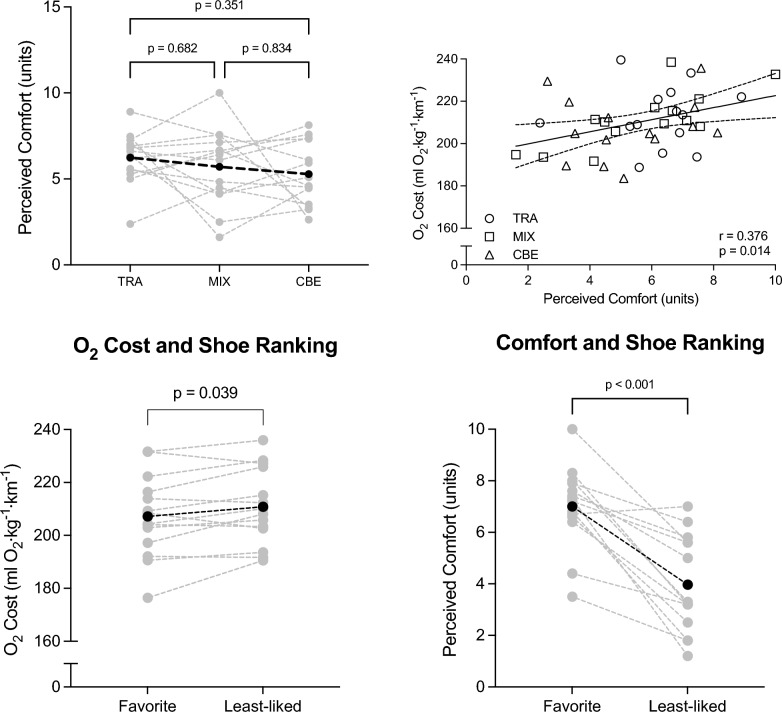
*Top left* Perceived comfort for the different footwear tested. *Top right* linear corelation between perceived comfort and O_2_ cost. *Bottom left* O_2_ cost according to most and least liked footwear on testing day 3. *Bottom right* Perceived comfort according to most and least liked footwear on testing day 3. *Grey lines* are individual values; *black line* is average. TRA = On Cloudrunner 2; MIX = On Prototype; CBE = On Cloudboom Echo 3

No significant effect of footwear was detected for perceived comfort (*p* = 0.377). Individual and average values for perceived comfort are displayed in Fig. 3. A positive association between perceived comfort and O_2_ cost was detected when all footwear conditions were plotted together (r = 0.376, *p* = 0.014, Fig. 3). However, when the data was analysed using repeated measures correlations, respecting the dependency of the three footwear trials for each participant, no significant correlation was detected (rrm_(27)_ = 0.11, 95% CI −0.271 to 0.455, *p* = 0.583, see Figure in Supplementary material).

Using the data from Visit 3, when participants were the most familiar with all the available footwear conditions, a two-tailed paired t-test analysis was performed to compare the O_2_ cost, as well as the perceived comfort, from the favourite ranked shoe with the least-liked shoe (Fig. 3). O_2_ cost when wearing the least-liked shoe was 3.5 ± 5.8 ml O_2_ kg^−1^ km^−1^ higher compared with the preferred shoe (95% CI: 0.2 to 6.8 ml O_2_ kg^−1^ km^−1^, *p* = 0.039, ES = 0.6). Comparison of perceived comfort of the favourite and least-liked shoes indicated a difference of 3.0 ± 1.9 units (95% CI: –−4.1 to −1.9 points, *p* < 0.001, ES = 1.6) in favour of the preferred shoes.

### Spatiotemporal Variables

Spatiotemporal data for the different footwear and speeds tested is presented in Table 2. Running cadence was mostly independent of footwear and speed, except for a higher cadence for CBE compared with MIX at 12 km h^−1^ (0.8 ± 1.0 steps min^−1^_,_ 95% CI 0.0 to 1.5 steps min^−1^, *p* = 0.039). No effects of footwear or shoe-speed interactions were detected for ground contact time, leg stiffness, flight ratio or impact magnitude. Impact duration was lower for CBE compared with TRA at both 7.5 km h^−1^ (2.7 ± 3.5 ms_,_ 95% CI 0.1 to 5.1 ms, *p* = 0.042) and 9.0 km h^−1^ (2.5 ± 2.8 ms_,_ 95% CI 0.4 to 4.5 ms, *p* = 0.018).

## Discussion

The aim of this study was to determine the effects of AFT shoes on O_2_ cost, spatiotemporal and perceptual variables at slow running speeds. While the AFT shoes provided the expected metabolic savings compared with traditional running shoes, in contrast to current literature and our own hypothesis the differences in running economy persisted even when testing at 7.5 km h^−1^, with no signs of a speed-dependent effect. Our second hypothesis was likewise not supported by our results, as no relevant differences in spatiotemporal variables were detected between the footwear conditions that could explain the differences in O_2_ cost between the different shoes. Finally, although a weak correlation between perceived comfort and O_2_ cost was found between individuals, it went in the opposite direction from what was expected, as higher comfort rating was associated with a higher O_2_ cost, which theoretically would not be beneficial for the runner. Nonetheless, shoe preference by participants seems to have been more strongly affected by perceived comfort than running economy.

The difference in running economy between the AFT and TRA shoes reported in the present study is in line with our previous investigation of the same shoes at 16 km h^−1^ with well-trained runners (2.4 ± 1.1%) [13]. In the present study O_2_ cost improved between 2.3 and 3.1% comparing CBE and TRA over the tested speed range. These findings, however, are in stark contrast with previous suggestions that the benefits of AFT shoes disappear at lower speeds [9, 10]. Both previous studies, however, used either single [10] or two [9] runs per speed and shoe tested, which could have increased results variability [25]. In the present study, even though the average difference between CBE and TRA was relatively similar considering day 1 only (2.9%) or all 3 testing days (2.5%), the effect size was drastically reduced when considering day 1 only (from d_z_ = 2.5 with all three trials down to d_z_ = 0.5 for day 1 only), due to increased variability in responses. Therefore, the present results also argue for repeated trials to reduce variability in footwear research. A lack of familiarization could also explain the differences in perceived effort between TRA and CBE only manifesting at the higher speeds of 10.5 and 12.0 km h^−1^. Most of the participants never wore AFT shoes prior to this study and any physical or mental effort required to adapt their running style to the shoes could have overshadowed the benefits of a lower O_2_ cost at lower speeds. Nonetheless, the absence of detectable changes in perceived exertion could lead to the question of whether differences in O_2_ cost alone would be enough to elicit performance improvements in this population.

Interestingly, the differences in O_2_ cost between shoes persisted even when comparing the CBE shoe to the more similar MIX shoe. A weight-matching between the MIX and CBE shoes could theoretically decrease the O_2_ cost benefits of the CBE to 0.6–1.3%, as the MIX shoe is 97 g heavier and O_2_ cost is expected to rise by approximately 1% for each 100 g of added shoe mass. This differs from the findings of Hébert-Losier et al. [11], who could not detected differences between a pair of AFT shoes and a pair of racing flats at 11 km h^−1^, which despite likely having lower energy return and bending stiffness was 58 g lighter. The MIX shoes, in fact, offered greater energy return compared with the CBE shoes, although through a combination of higher deformation and lower resilience. Softer shoes with better energy return properties do not necessarily improve running economy, as their lower-density foam requires increased thickness and compliance to store a similar amount of energy, resulting in greater compression and energy loss. The increased energy return compensates for this energy deficit but may not directly enhance running economy [30, 31]. On the other hand, the MIX shoes have lower longitudinal bending stiffness compared with the CBE shoes. Higher bending stiffness can improve running economy by providing a more efficient energy return during push-off [26, 27], although evidence also exists for negative effects of increased bending stiffness on running economy at slow speeds [27]. In any case, the consistent improvements in O_2_ cost found the comparing the CBE against both the TRA and the MIX shoes from 7.5 to 12 km h^−1^ do not support the existence of either a detrimental effects of high bending stiffness at low speeds, or the existence of a force threshold for AFT gains, as has previously been suggested [9].

Despite the differences in O_2_ cost and perceived effort between shoes, no meaningful differences in spatiotemporal metrics were detected, including ground contact time, leg stiffness, and measures of impact magnitude. While there were statistical significances in cadence between the MIX and CBE shoes at 12 km h^−1^, we note that these were very small in magnitude, and in fact smaller than other pairwise comparisons at other speeds. Therefore, it is difficult to attribute a more concrete meaning to these differences, especially given the consistent differences in O_2_ cost between shoes over the different speeds tested. Literature on the effects of AFT shoes on spatiotemporal variables is ambiguous, with no clear changes in spatiotemporal metrics being effectively associated with differences in O_2_ cost [1, 4, 10]. This does not mean that biomechanical explanations for the differences between footwear conditions should be disregarded: a bigger variability in gait parameters and longer adaptation period to new footwear of recreational runners have been reported in the past [28], and could also be a factor in the present study. In this regard, we note that the device used here for assessing spatiotemporal variables has a typical error of under 1.5% for cadence and ground contact time, whereas that for impact magnitude and duration is higher, between ~3 and 6% [13]. Finally, as many participants had limited experience with treadmill running, they potentially adopted different gait mechanics than they would outdoors [29]. As only a small difference in impact duration between the CBE and the MIX shoes were detected at the lower speeds, more research is needed to elucidate whether this would bring any functional difference to runners, for instance in terms of injury risk [30], as a shorter impact duration suggests a higher rate of loading for the lower limbs. Although higher loading rates are often suggested as potential modifiers of injury risk, the relationship between the two remains unclear [31].

Due to substantial variations in the opinions of recreational runners, no significant effects of footwear condition on perceived comfort were found. A potential effect of running speed on the influence of longitudinal bending stiffness on subjective comfort was suggested in a previous study, as a higher stiffness seems to be preferred at faster running speeds [32], because high longitudinal bending stiffness can hinder the natural foot movement and reduce comfort at lower running speeds [33]. Taking this into account, potential effects between the footwear conditions could have been diminished as the participants provided a single overall comfort rating at the end of each run, reflecting their experience across all four tested speed stages. It is possible that although the higher longitudinal bending stiffness of the CBE shoes may not have been perceived as comfortable at the slower speeds, this did not interfere with the benefits in O_2_ cost. Still, a significant relationship was also detected between comfort and O_2_ cost when all shoes were analysed together, which unexpectedly went against our proposed hypothesis, namely that higher comfort would be associated was lower O_2_ cost. On the other hand, when repeated measures correlation for the three footwear conditions were performed, no correlation between perceived comfort and O_2_ could be established. Another factor to consider is that most of the participants had no experience with AFT shoes prior to this study: a potential influence of footwear habits, meaning that the closer the shoes are to their habitual shoes, the more they are perceived as comfortable, needs to be considered in future studies [33]. Interestingly, the overall preferred shoe ranking from day 3 revealed that the favourite shoe not only had better comfort but also demonstrated a lower O_2_ cost compared with the least preferred shoe. However, the effect size of the differences in comfort was considerably larger than that of O_2_ cost. This suggests that even though performance considerations factor into the process of shoe choice, perhaps in some form of a shoe “feeling fast”, subjective comfort plays a more dominant role, reflecting the complexity of the decision-making processes in shoe choice.

### Limitations

Our study is not without limitations. On the one hand, the duration of the tests was short, at 3 min per speed. Given that the speeds were tested continuously, steady state was reached within the discarded window of the tests, and the remaining period (80 s) is well within what is adopted in the literature. However, it could have been beneficial to test speeds faster than 12 km h^−1^. In our cohort, however, most participants would struggle to finish a stage at 13.5 km h^−1^, as they were in the lower end of the inclusion criteria for running performance.

Another aspect that needs mention is the lack of weight matching of the shoes. As features like increased midsole thickness and upper padding for added energy return or comfort cannot be implemented without incurring an increase in shoe mass, we opted for not matching the different models used. For every 100 g of additional shoe mass, the running economy is impaired by ~1% [34, 35]. For instance, given that the TRA shoes are 62 g heavier than the CBE shoes, O_2_ cost could be expected to improve by 0.6% due to shoe mass alone. However, this would still yield a difference of 1.7–2.5% difference in O_2_ cost for the different speeds that must be explained by features other than shoe mass.

In this context, it also must be taken into consideration that a higher degree of variability is present in the gait of recreational runners compared with better trained individuals [28], which reduces statistical power. However, as the purpose of this study was to investigate the effects of AFT shoes during slow running, it was decided that runners who actively trained and competed at those speeds should be recruited, or else results could be biased from selecting well-trained individuals. We tried to overcome this issue by performing multiple trials per participant, which should decrease variability.

Lastly, the ecological validity of this study was impacted by testing indoors on a motorized treadmill, as previous studies have demonstrated differences in running biomechanics between laboratory and outdoor running [29, 36, 37]. As our cohort consisted of recreational runners, which are often not used to pace themselves as well as better trained individuals, we considered that the adverse effects of inconsistent speeds on a track or road would outweigh the lost ecological validity created by the indoor testing.

## Conclusion

In conclusion, AFT shoes can provide reduction in O_2_ cost of running over a large range of slow running speeds. The metabolic savings appear to be independent of speed and are robust enough to manifest as lower heart rate and even perceived effort at the higher speeds tested. The metabolic differences found do not seem to be supported by changes in spatiotemporal variables. Further research is needed to assess the suitability of AFT shoes for this specific population, particularly in relation to injury risk and training recommendations.

While the precise mechanisms underlying the improvements in running economy from use of AFT shoes remain unclear and require further investigations, the present study shows that prioritizing compliance and energy return does not necessarily lead to improved O_2_ cost and perceived comfort in recreational runners, who seem to prioritize the latter in their footwear choice. A more suitable approach for recreational runners might involve a light-weight design that balances moderate bending stiffness and compliance with regulated deformation and high resilience, thereby enhancing both comfort and running economy.

## Supplementary Information

Supplementary Material 1.

## Data Availability

The primary data used for this study is available upon reasonable request to the corresponding author.
