# Supplementary material for: Impact of Advanced Footwear Technology on Running Economy at Slower Running Speeds: A Randomised, Cross-Over Investigation
Source: Sports Med Open. 2026 Feb 17;12:12. doi: 10.1186/s40798-026-00977-3 (PMC12913831; doi:10.1186/s40798-026-00977-3)
Supplement: Supplementary file 1 — Supplementary Material 1. [file 40798_2026_977_MOESM1_ESM.docx]

Supplementary Information

Journal: Sports Medicine – Open

Title: Impact of Advanced Footwear Technology on Running Economy at Slower Running Speeds: a randomized, cross-over investigation

Authors: Aline Bolliger ^a^, Christina M. Spengler ^a^, Fernando G. Beltrami ^a^

^a^ Exercise Physiology Lab, Institute of Human Movement Sciences and Sport, ETH Zurich, Zurich, Switzerland

A series of perceptual questions were aked to participants in an attempt to contextualize the perception of comfort and overall satisfaction with the the different shoes. We acknowledge that these scales have not been validated. They may nonetheless provide valuable insights for the construction of future questionnaires that better encapsulate the runner’s experience with different footwear.

Here we provide the specific questions asked to the participants and the graphical representation of responses.

Question: How does the footwear affect your running form?

(0 = does not affect at all, 10 = maximal imaginable effect)

Question: Does the shoe feel springy, i.e. does it feel like it gives back energy?

(0 = no energy return, 10 = maximal imaginable energy return)

Question: How suitable does this shoe feel for the range of speeds you performed?

(0 = not at all suitable, 10 = perfectly suitable)

Question: How does this shoe fit your feet?

(0 = does not fit at all, 10 = perfect fit)

Question: Overall, how satisfied are you with this shoe?

(0 = not at all satisfied, 10 = completely satisfied)

A repeated measures correlation was performed between perceived comfort and O_2_ cost (three ratings per participant, one for each footwer condition). In the plot below, each participant is rpresented with a different colour. No significant correlation was detected (rrm_(27)_ = 0.11, 95% CI -0.271 to 0.455, p = 0.583).
